# A Mini-Review on Ceftaroline in Bacteremia Patients with Methicillin-Resistant *Staphylococcus aureus* (MRSA) Infections

**DOI:** 10.3390/antibiotics8010030

**Published:** 2019-03-20

**Authors:** Nicole Lounsbury, Mary G. Reeber, Georges Mina, Christiane Chbib

**Affiliations:** 1 Department of Pharmaceutical Sciences, Larkin University College of Pharmacy, 18301 North Miami Ave, Miami, FL 33169, USA; nlounsbury@ularkin.org (N.L.); mreeber@myularkin.org (M.G.R.); 2 Pharmacy department, Jackson Memorial Hospital, 1611 NW 12th Ave, Miami, FL 33136, USA; bicnu@hotmail.com

**Keywords:** ceftaroline, cephalosporin, methicillin resistant *Staphylococcus aureus* (MRSA), pediatrics, safety, community-acquired infection, bacteria infection, pneumonia, skin infection, MRSA bacteremia

## Abstract

Objective: The objective of this review is to describe the outcomes of patients treated with ceftaroline in the non-Food and Drug Administration (FDA) approved indication of methicillin-resistant *Staphylococcus aureus* (MRSA) infections in both pediatric and adult populations. Data sources: A systematic overview was conducted by searching PubMed, Medline, and The Cochrane Library up to January 2019. Study selection and data extraction: All English-language clinical trials and case reports related to the efficacy of ceftaroline in new, not-yet-approved FDA indications in MRSA infections in pediatric or adult populations. Data synthesis: In the case of MRSA bacteremia (MRSAB) infections, three different randomized studies in pediatric patients showed effectiveness of ceftaroline. When used in the case of adult populations with MRSA bacteremia, a small trial of 16 patients showed 50% clinical success in patients with acute bacterial skin and skin structure infections versus 63% clinical success in patients with community-acquired bacterial pneumonia. Another case series of six refractory case reports showed 50% clinical success of ceftaroline in patients with MRSA. Conclusions: Although there are few case reports and limited data to date, ceftaroline fosamil should continue to be studied as an alternative therapy in MRSA infections in both pediatric and adult populations. Clinical success rates of ceftaroline were, in most cases, considered high when treating patients with MRSA infection. More clinical trials need to be studied. In the specific case of MRSA bacteremia, the treatment options remain few and ceftaroline should be extensively studied for the salvage treatment of MRSAB.

## 1. Introduction

Ceftaroline is a novel cephalosporin, given to patients by intravenous (IV) infusion. It is the active form of ceftaroline fosamil, a bactericidal antibiotic with Gram-positive and -negative coverage. Ceftaroline fosamil, branded as Teflaro^®^ (Forest Laboratories, Inc., New York, NY, USA) in the United States, was approved by the Food and Drug Administration (FDA) in 2010 for adults and in 2016 for children older than two months for two indications: Complicated skin and soft tissue infections (cSSTI) and community-acquired pneumonia (CAP) [1]. Ceftaroline showed superior efficacy to ceftriaxone in adults with CAP in two phase 3 trials: FOCUS 1 and FOCUS 2 [2,3,4]. They were multi-centered, multinational, randomized trials which evaluated the safety and efficacy of 600 mg intravenous (IV) every 12 h ceftaroline fosamil compared to ceftriaxone 1 g IV every 24 h for 5 to 7 days for treatment of hospitalized CAP patients, but did not include patients with methicillin-resistant *Staphylococcus aureus* (MRSA) infections. In this update, we will discuss new applications of ceftaroline for the treatment of MRSA in both adult and pediatric populations based on new case reports, clinical trials and other observational studies reported in literature. This review will mainly include: (1) the discussion of the mechanism of action of ceftaroline, (2) the antimicrobial activity against *S. aureus* pathogens isolated from the patients included in this review and (3) the literature updates on ceftaroline use in pediatric and adult population with both MRSA infections in general and MRSA bacteremia.

## 2. Data Sources

A systematic overview was conducted by searching PubMed, Medline, The Cochrane Library reports published up to January 2019. The search terms used include ‘’ceftaroline’’, ‘’adult methicillin resistant Staphylococcus aureus’’, ‘’pediatrics’’, ‘’pediatric methicillin resistant *Staphylococcus aureus*, bacteremia’’ and antimicrobial activity. The studies selected in this review are the ones that provide data for the use of ceftaroline in patients with MRSA infections. Most of the reported studies in literature assess the use of ceftaroline in patients with MRSA bacteremia.

## 3. Chemistry/Mode of Action/Pharmacology

### 3.1. Chemistry and mode of action of ceftaroline

**Figure 1:**
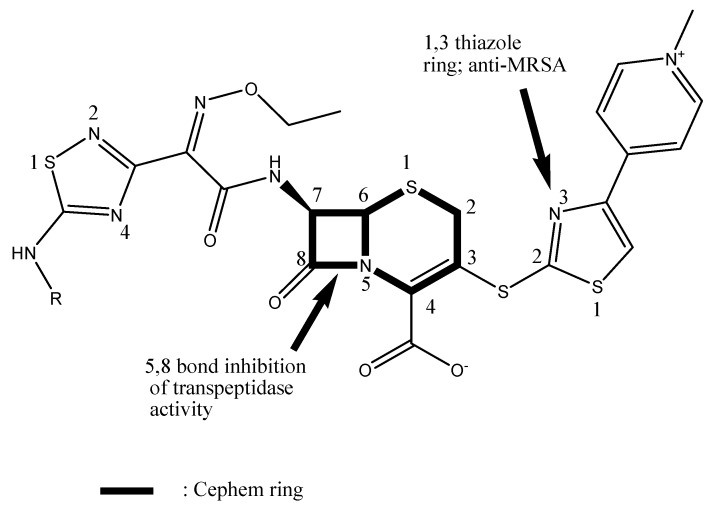
Structure–activity relationships for active ceftaroline (R=H); the prodrug ceftaroline fosamil has the phosphono group at R = −P(=O)(OH)_2_ [11].

Ceftaroline is described as a “fifth-generation” cephalosporin due to its reported broader activity against Gram-positive bacteria such as MRSA [5]. Its anti-MRSA activity is related to the addition of the 1,3-thiazole ring moiety to its structure. Ceftaroline exerts a bactericidal effect through inhibition of the bacterial cell wall synthesis [6,7]. This is achieved by its binding to the penicillin-binding proteins (PBPs), including PBP2a (which confers resistance to MRSA) and PBP2x (which confers resistance to penicillin-resistant *S. pneumoniae*) [8]. Ceftaroline causes a conformational change in PBP2a which allows binding to the active site of the protein [9]. The activity of ceftaroline against MRSA is due to its 1,3-thiazole ring on the 3rd position of the cephalosporin and the oxime in the acyl group attached to the 7th position of the cephalosporin [10] (Figure 1). The ability to penetrate Gram-negative bacteria is due to the increase in affinity to the transpeptidase enzyme caused by the 1,2,4-thiadiazole ring of the 7th position of the cephalosporin. To increase water solubility, a phosphono group was added, leading to the prodrug ceftaroline fosamil [10] in the form of acetate. Its active metabolite is ceftaroline which lacks the phosphono group present in the prodrug [11,12] (Figure 1).

### 3.2. In Vitro Antimicrobial Activity of Ceftaroline against S. aureus

Based on the breakpoints of ceftaroline against *S. aureus* followed by the European Committee on Antimicrobial Susceptibility Testing (EUCAST), MIC > 1 mg/L is counted as resistant. Whereas, following the breakpoints of the FDA, MIC = 2 mg/L is counted as intermediate and MIC > 2 mg/L is counted as resistant. Two studies reported the isolation of MRSA microorganisms from pediatric patients in literature and their MIC ranges show to be considered susceptible to ceftaroline (Table 1) [13,14]. Based on this evidence, more research needs to be conducted to evaluate ceftaroline susceptibility testing against isolated MRSA strains in pediatrics.

## 4. Specific Populations

### 4.1. Ceftaroline Use in Pediatrics MRSA

Ceftaroline fosamil was approved by the FDA in 2016 for pediatric patients from 2 months to 18 years of age to treat two specific indications: Acute bacterial skin and skin structure infection (ABSSSI) (for methicillin sensitive *Staphylococcus aureus* MSSA and MRSA pathogens) and community-acquired bacterial pneumonia CABP (for only MSSA). Recent analyses have shown that community-acquired MRSA (CA-MRSA) first occurred in children in the United States instead of adults [15]. A systematic overview was conducted by searching PubMed, Medline, The Cochrane Library and other reliable outlets up to January 2019. Reports of pediatric patients with MRSA infections are documented but no clinical trials exist yet. In this manuscript, we report two studies that evaluated the use of ceftaroline in patients with MRSA infections (MIC = 0.5–1 µg/mL). Even though the number of patients assessed is low, some clinical response and stability was shown after the use of ceftaroline in some patients. Korczowski et al. [13] evaluated the use of ceftaroline in pediatric patients with ABSSSI. Clinical success at day 3 was defined as: ≥20% reduction in total infection area and cessation of spread by length and width and temperature ≤37.6 °C. Clinical cure was defined as total resolution of all signs and symptoms. In a second study conducted by Blumer et al. [16], which evaluated ceftaroline’s safety and effectiveness compared to ceftriaxone plus vancomycin in pediatric patients with complicated CABP [17], clinical response was defined as an improvement in at least two and worsening in none of the following seven symptoms: Cough, dyspnea, chest pain, sputum production, chills, fever and lethargy or exercise intolerance. Clinical stability was defined as being afebrile, normal pulse and respiratory rates, ≥92% oxygen saturation and worsening of none of the seven symptoms stated previously. The percentage of clinical success fluctuated: Korczowski et al. reported it as 50% while Blumer et al. reported it as 89% (Table 2) [13,16]. In 2018, a case report [17] described a pediatric patient with a persistent MRSAB associated with cellulitis, fasciitis, myositis, and a deep vein thrombosis causing septic pulmonary emboli. This condition was unsuccessfully treated with vancomycin monotherapy first, then daptomycin monotherapy afterwards (vancomycin: MIC = 2 mg/L; daptomycin: MIC = 1 mg/L). After nine days of treatment, ceftaroline (MIC = 0.75 mg/L) was added to the latter antibiotic for a period of four weeks. It was reported to have cleared the bacteremia with proven clinical improvement. This fact warrants the instigation of further randomized controlled trials with a larger number of patients.

### 4.2. Adults MRSAB

Upon searching the literature, no randomized controlled trials exist for the treatment of MRSAB with ceftaroline. Results from the 2010 AWARE study, which evaluated antimicrobial resistance, showed that ceftaroline has high activity in vitro against MRSA isolates collected from different medical centers in the US [14]. Based on the 2011 Infectious Diseases Society of America (IDSA) MRSA guidelines, vancomycin or daptomycin are still the treatment of choice for both complicated and uncomplicated MRSAB [18,19]. No agent has proven to be superior to vancomycin or daptomycin, but in the case of persistent MRSAB, data of different case reports or small subgroups of clinical trials prove the basis for alternate agents including quinupristin-dalfopristin, trimethoprim-sulfamethoxazole, linezolid, and telavancin [18,19]. In this review, we collected data found in literature that assessed the use of ceftaroline in adult patient population with MRSA infections (MIC = 0.5–1 µg/mL) (Table 3) [20,21,22,23,24,25,26]. The clinical success of ceftaroline in MRSAB patients, reported in Table 3, varied between 50% and 88%. This report indicates the need to initiate further randomized controlled trials with a larger number of patients to increase the evidence for treatment options in the adult population. A systemic review and meta-analysis was conducted by Sotgiu and coworkers [27] for efficacy/effectiveness-related outcomes of ceftaroline in patients with pneumonia. The overall efficacy/effectiveness of ceftaroline was 81.2% in all types of pneumonia. Specifically in MRSA cases, success rates were documented as 71.7%. Additionally, the use of ceftaroline in Gram-positive osteomyelitis was studied by Johnson and co-workers [28]. MRSA pathogens were isolated in 94/150 patients (62.0%) in a phase 4 clinical assessment program. Out of 93 patients with MRSA pathogens receiving ceftaroline therapy, 86 patients showed clinical success (92.5%) defined as discontinuation of ceftaroline following clinical cure with no additional need of antibiotics or switch to another antibiotic. Ceftaroline has also been studied by Destache and coworkers [29] in the treatment of Gram-positive endocarditis. In this study, out of 55 patients, 44 (80%) had isolated blood MRSA pathogens. When given ceftaroline as a monotherapy, clinical success was observed in 19 out of 23 patients (82.6%). When used as first or second line, the clinical success achieved was 75.0% and 70.6% respectively. The patients with MRSA in this study, have exhibited a total of 77.3% (34/44) clinical success rate. This report provides growing evidence of the use of ceftaroline in adult populations with MRSAB infections.

Sakoulas et al. [25] described finding in vitro synergy where ceftaroline was shown to induce daptomycin binding in MSSA and MRSA which could be an opportunity to hasten clearance of refractory staphylococcal bacteremia. In an effort to optimize the therapeutic option in MRSA patients, Shafiq et al. [30] studied two clinical isolates from a 68-year-old patient with MRSAB. It was found that the combination of ceftaroline and daptomycin resulted in reduction of time-kill experiments against MRSA isolates. Further research of the in vitro synergy with daptomycin and ceftaroline combination was found to be caused by multiple mechanisms: a decrease in bacterial cell-wall thickness, an increase in daptomycin binding and an increase in daptomycin-induced depolarization [24,31,32]. In 2014, a retrospective, multicenter, study included 23 cases of refractory staphylococcal bacteremia that persisted for a median of 10 days, while the patients were on appropriate therapy regimens. Bacteremia was reported to be cleared in a median of two days after receiving the combination of daptomycin and ceftaroline [25]. It may be a worthwhile addition to daptomycin in refractory cases. In an effort to study further daptomycin-ceftaroline synergism, Cortes-Penfeld and coworkers [33] performed a retrospective chart review of patients with MRSAB after receiving treatment with vancomycin in combination with ceftaroline. Seventeen patients were included in the study: The first group consisted of four patients who received the combination as a second line therapy. The second group of eight patients received the combination as a third line therapy and the third group of five patients received daptomycin alone. This study suggests that 2nd line therapy rather than 3rd line with the combination of daptomycin and ceftaroline resulted in a shorter duration of bacteremia (6.8 vs. 11.5 days; *p* = 0.08) but there was no difference in the rates of mortality (75% vs. 62%; *p* = 1.0). It is worthy to note that patients who received daptomycin alone had 20% mortality which is lower than the combination of both groups 1 and 2: 62.5% and 75%, respectively. This scientific fact should trigger further evaluation of ceftaroline in combination with other antibiotics. Many questions arise regarding which combination has led to a faster cure clinically and microbiologically and whether ceftaroline should be given as a combination as well as whether ceftaroline is more efficient in refractory cases, even though there are insufficient data to prove it.

## 5. Conclusions

MRSA is known to cause a variety of infectious problems including ABSSSIs, and skin and soft tissue infections in adults and children. Alternative medications must be developed to treat patients who cannot be treated with traditional therapies such as vancomycin and clindamycin. Although there are few case reports and limited data to date, ceftaroline fosamil should continue to be studied as an alternative therapy for patients with MRSA. Ceftaroline fosamil is a broad-spectrum cephalosporin antibiotic that has been used to treat against MRSA in refractory cases. Ceftaroline fosamil has been shown to be safe and well-tolerated among children and adults, including those with MRSA, who are resistant or require an alternative antibiotic to common treatments. Its efficiency still needs to be studied extensively. Since the treatment of bacteremia might extend to over 14 days, hematological complications like neutropenia need to be assessed. The co-administration of ceftaroline and daptomycin in MRSAB has shown to be effective, but more clinical data needs to be evaluated and studied. Ceftaroline’s place in the treatment regimen of pediatric and adult population with MRSA infection needs to be determined.

*Key points:*

- Some evidence in literature suggests that ceftaroline used for the treatment of MRSA has been shown to be successful in some cases in terms of clinical cure.
- A combination of ceftaroline and daptomycin has shown to be successful in treating patients with MRSA infections in both adult and pediatric populations.
- A synergy mechanism was observed in vitro when ceftaroline was added to daptomycin therapy. Clinical evidence of the benefits of the combination of the two drugs still needs to be thoroughly studied.
- There is still limited data to date regarding the efficacy of ceftaroline alone or when compared to other antibiotics for the treatment of MRSA like quinupristin-dalfopristin, trimethoprim-sulfamethoxazole, linezolid, and telavancin.
- Evidence suggests that ceftaroline has been shown to be safe when administered in both adults and pediatrics.

## Figures and Tables

**Table 1:** MIC 50/90 of ceftaroline for MRSA in pediatric patients.

Study	MIC 50/90 of Ceftaroline for MRSA
Multicenter, randomized, observer-blinded, active-controlled in pediatrics [13]	MRSA = 0.5/1 mg/L
AWARE study in pediatrics [14]	MRSA = 0.5/1 mg/L

**Table 2:** Reports of trials that used ceftaroline to treat MRSA infection.

Criteria	Korczowski et al. [13]	Blumer et al. [16]
Total number of patients included, *n*	159	5
Patients with MRSA, *n* (%)	18 (11%)	1
Patients who received antibiotics prior to ceftaroline	9	1
Duration of treatment with ceftaroline, median (range)	3 days to 10 days	-
Clinical success of MRSA patients, *n* (%)	16/18 (89%)	1
Safety outcome	8% diarrhea 8% rash 7% vomiting 1% pruritis Serious adverse effects reported: 1 patient experienced hypersensitivity and another clostridium difficile colitis No death reported	Anemia, pruritus and vomiting

**Table 3:** Summary of trials for the use of ceftaroline in adult patients with MRSA infection. (ABSSSI = Acute bacterial skin and skin structure infection; CABP = community-acquired bacterial pneumonia; SAB = *Staphylococcus aureus* bacteremia).

Criteria	Ho et al. [20]	Casapao et al. [21]	Vazquez et al. [22]	Lin et al. [23]	Polenakovitch et al. [24]	Sakoulas et al. [25]	Santos et al. [26]
Total number of patients who received ceftaroline, n	6	630	48 (27 with ABSSSI and 21 with CABP)	10	31	26	647
Patients with MRSA, *n* (%)	6 (100%)	241 (38%)	16 (59%) with ABSSSI and 16 (76%) with CABP	10 (100%)	31 (100%)	20 (76%)	191 (29%)
Patients who received antibiotics prior to ceftaroline	6	422	14 with ABSSSI and 13 with SAB	10	31	26	515
Duration of treatment with ceftaroline, median (range)	Varies per case	6 days	5.8 days for ABSSSI and 7 days for CABP	Varies per case	5 days	16 days	6 days
Number of patients that were treated with ceftaroline as monotherapy	6	447	22 in ABSSSI and 10 in CABP	-	-	none	114
Clinical success of MRSA patients, *n* (%)	5 (83%)	426/484 (88%)	8/16 (50%) with ABSSSI And 10/16 (63%) with CABP	6 (60%)	23 (74%)	23 (88%)	144/178 (81%)
Safety outcome	GI bleeding and death reported in one patient	8% hospital mortality 0.9% diarrhea 0.6% vomiting 1.1% renal failure	-	Rash, eosinophilia, pruritis and *clostridium difficile* infection	Eosinophilic pneumonia, rash and diarrhea	-	-
